# Supplementary material for: Biochemical principles of miRNA targeting in flies
Source: Nat Commun. 2026 Jan 20;17:1641. doi: 10.1038/s41467-026-68360-0 (PMC12905440; doi:10.1038/s41467-026-68360-0)
Supplement: Supplementary file 1 — Supplementary Information [file 41467_2026_68360_MOESM1_ESM.pdf]

Supplementary Information

Biochemical Principles of miRNA Targeting in Flies.

Vega-Badillo et al.

Supplementary Figure 1 | Enrichment profile of canonical sites in RBNS datasets generated in this study. Binding experiments were performed with 3.5 mM **(a)** and 0.89 mM **(b)** Mg^{2+}. Bottom panel: miRNA sequences used in Ago1 loading.

Vega-Badillo et al., Supplementary Figure 1

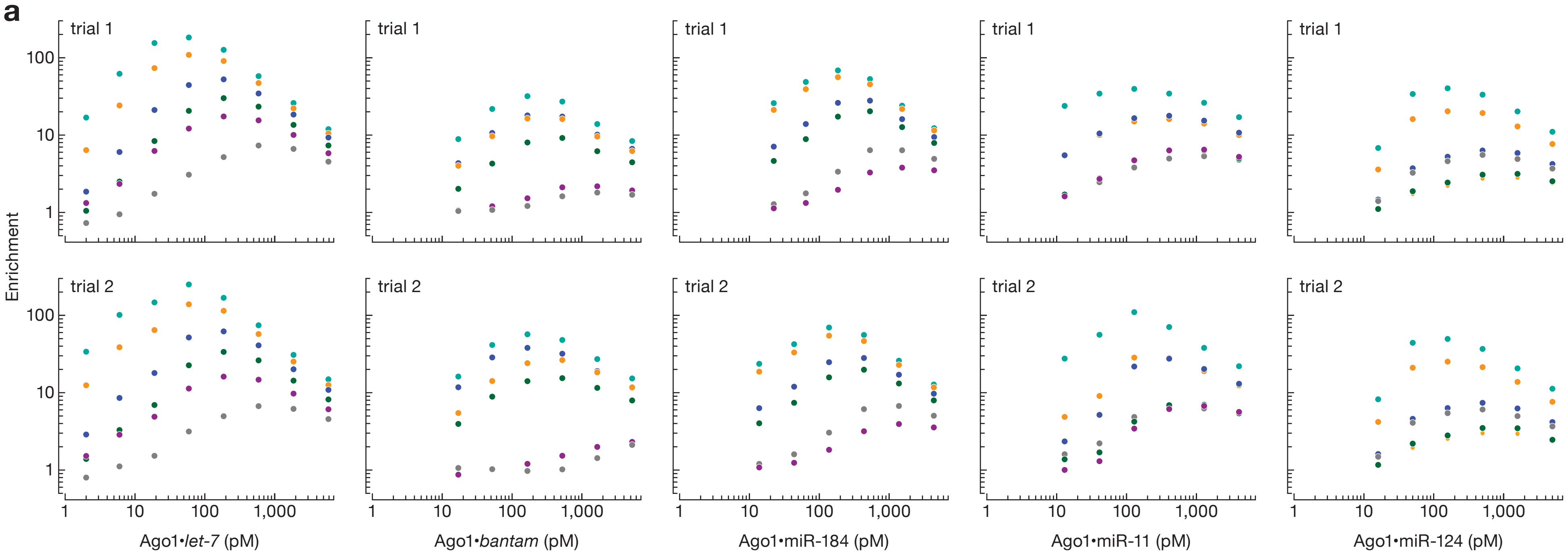

let-7
5′-UGAGGUAGUAGGUUGUAUAGU-3′

bantam
5′-UGAGAUCAUUUUGAAAGCUGAUU-3′

miR-184
5′-UGGACGGAGAACUGAUAAGGGC-3′

miR-11
5′-CAUCACAGUCUGAGUUCUUGC-3′

miR-124
5′-UAAGGCACGCGGUGAAUGCCAA-3′

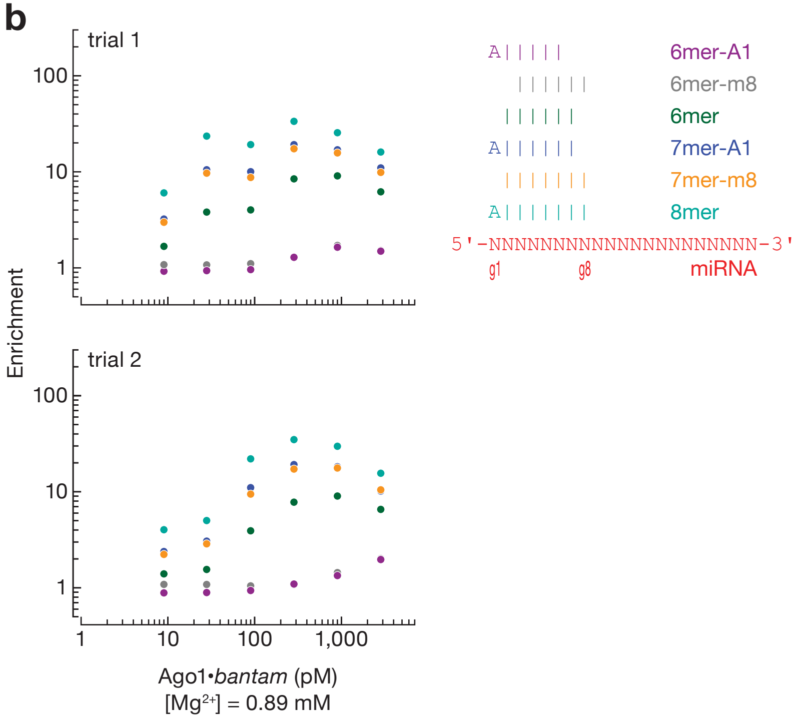

Supplementary Figure 2 | The effect of G:U pairs within the seed on miRNA binding affinity. (a) K_D values for canonical seed-matched sites (left: t2–t8 targets with t1A; right: t2–t8 targets) and one, two and three G:U pairs within *bantam*, miR-11, miR-184, and miR-124 seed at indicated positions. Shown for comparison: 8mer (cyan) and 7mer m8 (orange). Error bars indicate 95% CI on the median. B represents C, G, or U. **(b)** The equilibrium dissociation constant of *let-7*-loaded Ago1 for the competitor fully complementary to *let-7*, 30-nt poly(A) RNA and 30-nt poly(A) RNA, which contain no exocyclic groups and cannot pair with any natural base. miRNA sequence shown in red. Data are mean ± SD for three independent experiments. Source data are provided as a Source Data file.

Vega-Badillo et al., Supplementary Figure 2

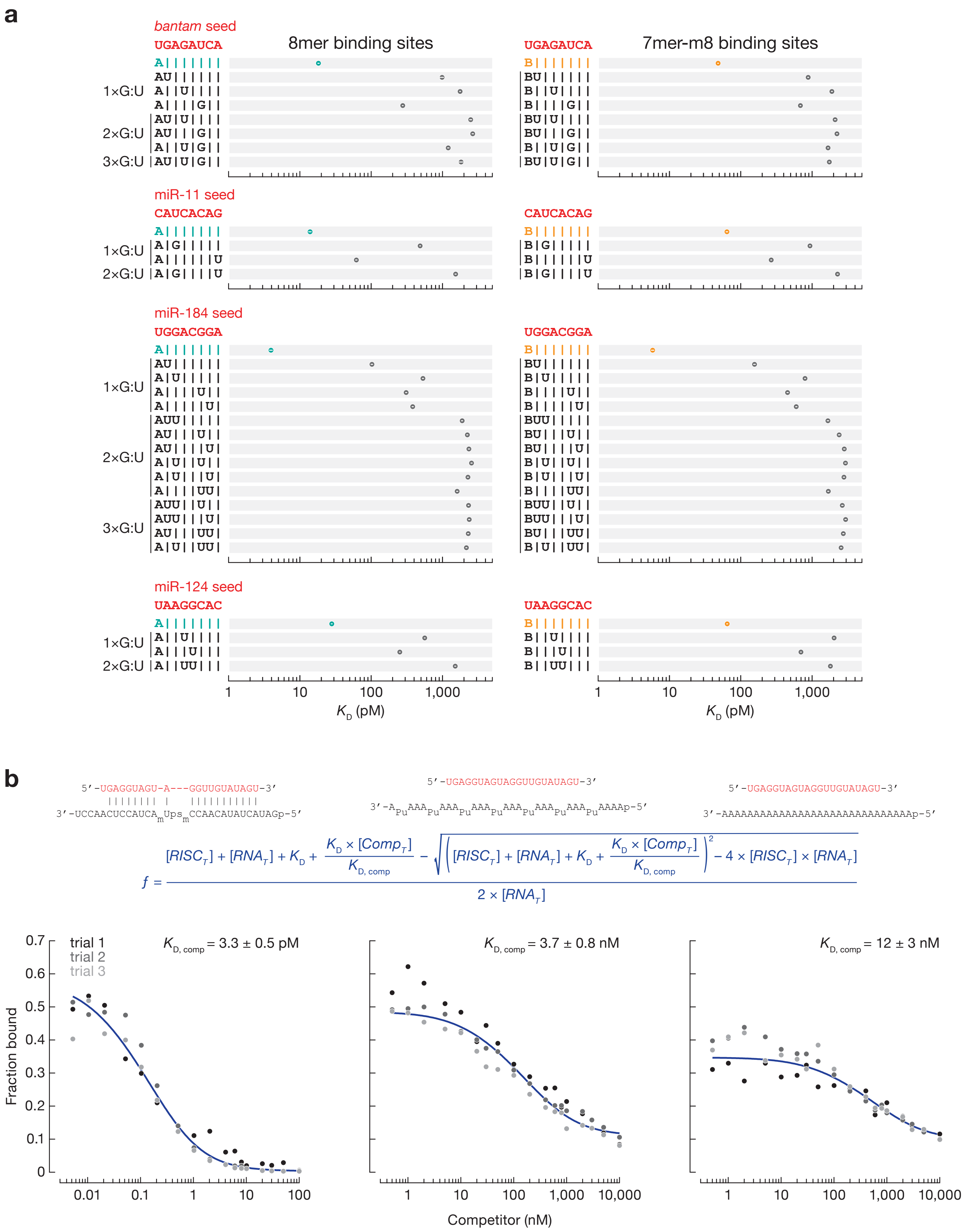

$$f = \frac{[RISC_T] + [RNA_T] + K_D + \frac{K_D \times [Comp_T]}{K_{D,\,comp}} - \sqrt{\left([RISC_T] + [RNA_T] + K_D + \frac{K_D \times [Comp_T]}{K_{D,\,comp}}\right)^2 - 4 \times [RISC_T] \times [RNA_T]}}{2 \times [RNA_T]}$$

Supplementary Figure 3 | K_D values for miR-124 t2–t8 targets with t1A and one-nucleotide mismatches **(a)**, deletions **(b)** and bulges **(c)** at indicated positions. Error bars indicate 95% CI of the median. Adenine (orange), uridine (magenta), cytidine (green), and guanosine (blue). miRNA g1–g8 sequence shown in red. **(d)** Comparison of binding affinities for the nucleation bulge sites and for 6mer-A1 sites with similar flanking context.

a

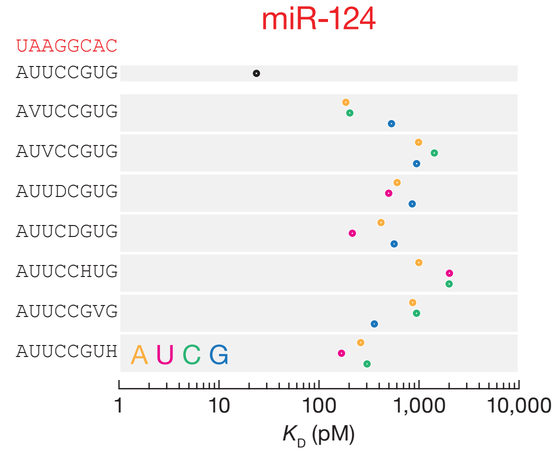

b

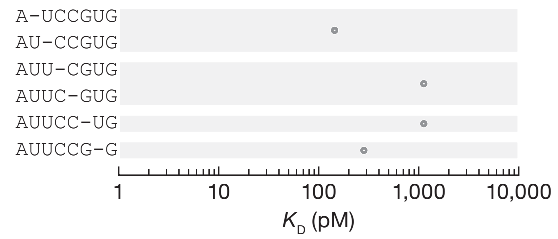

c

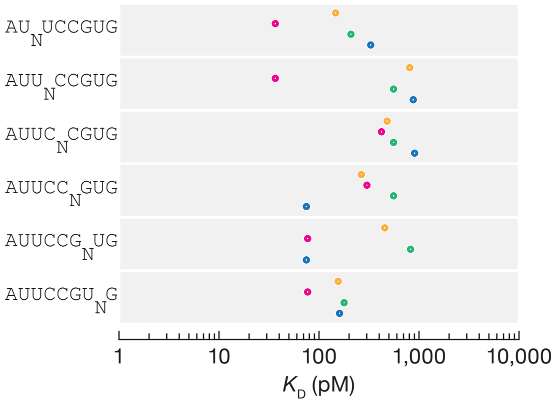

d

$$\Delta K_D \text{ (in pM)} = K_D^{\text{6mer-A1}} - K_D^{\text{nucleation bulge site}}$$

miRNA	median	95% CI
let-7	5.25	[3.60; 7.11]
bantam	444	[429; 460]
miR-11	169	[163; 175]
miR-184	699	[690; 707]
miR-124	701	[692; 713]

Supplementary Figure 4 | Nearest neighbor free energy values for t2–t8 targets with t1A and one-nucleotide mismatches **(a, b)**, deletions **(c, d)** and bulges **(e, f)** at indicated positions. Values for fly **(a, c, e)** and mammalian **(b, d, f)** miRNAs have been computed at 25°C and 37°C, respectively, from experimentally determined KD values using the formula $\Delta G = RT \ln(K_D)$. B represents C, G, or U; D represents A, G, or U; H represents A, C, or U; V represents A, C, or G. Adenine (orange), uridine (magenta), cytidine (green), and guanosine (blue). miRNA g1–g8 sequence shown in red. Horizontal dashed lines indicate the nearest neighbor free energy for t2–t8 targets with t1A.

Vega-Badillo et al., Supplementary Figure 4

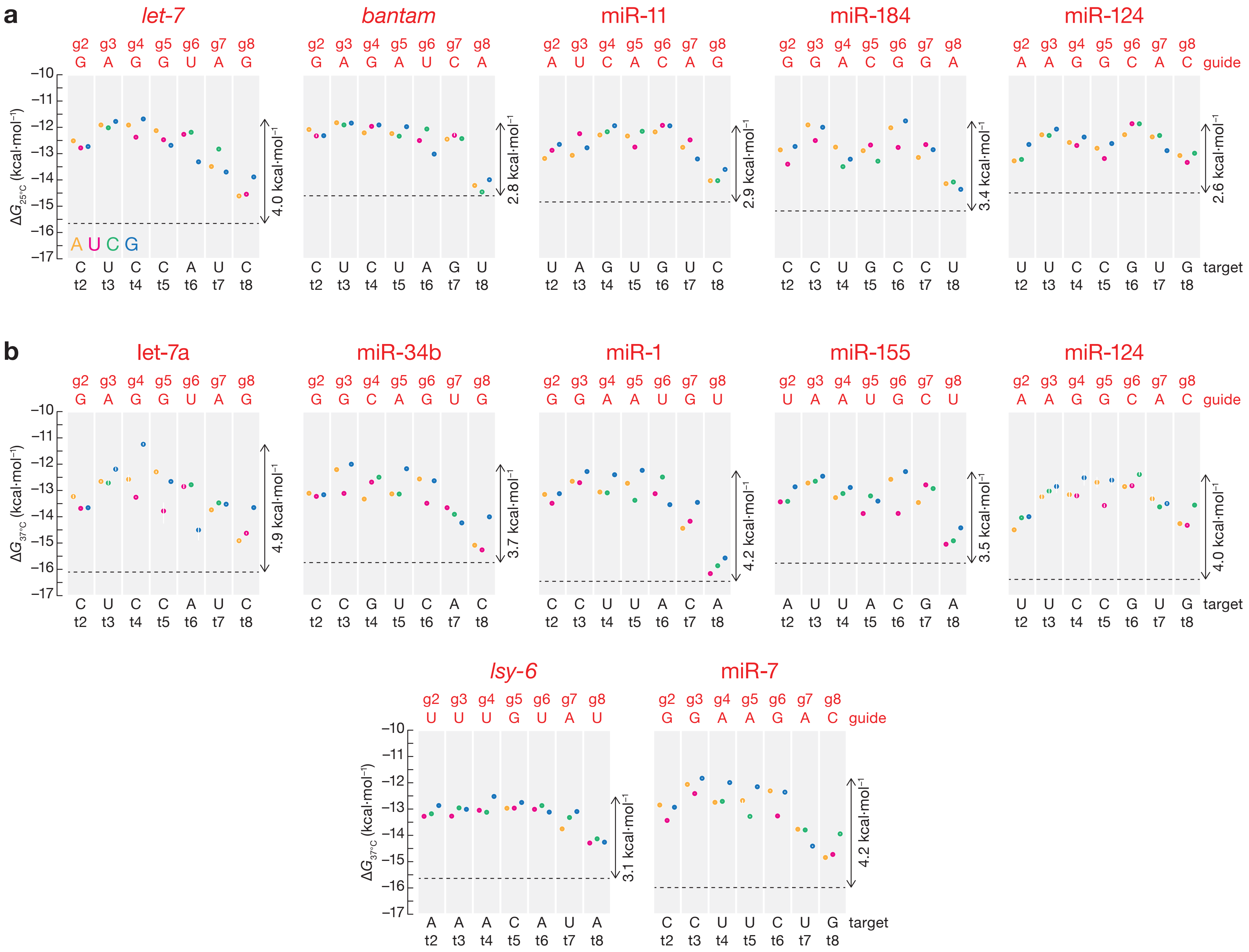

Vega-Badillo et al., Supplementary Figure 4

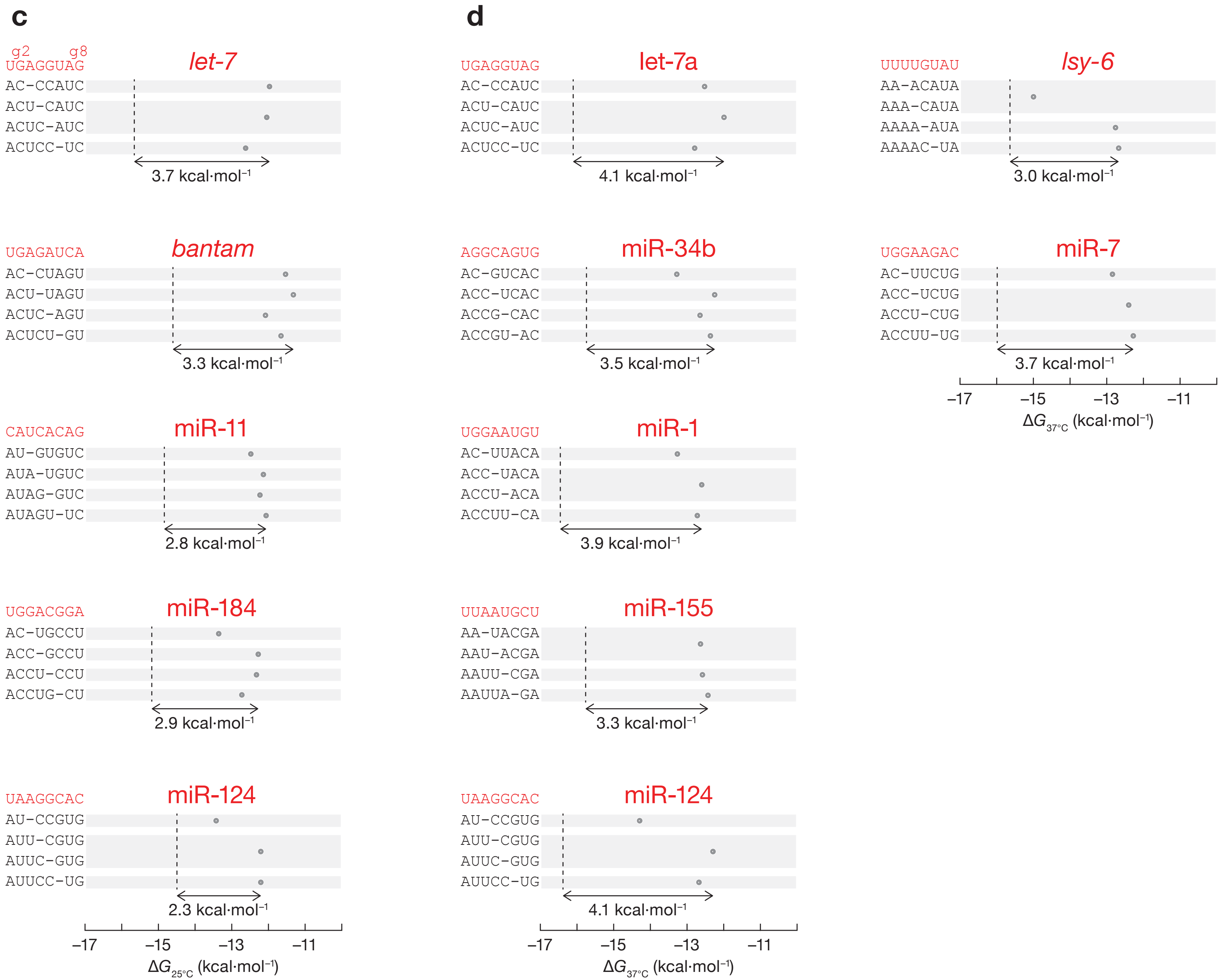

Vega-Badillo et al., Supplementary Figure 4

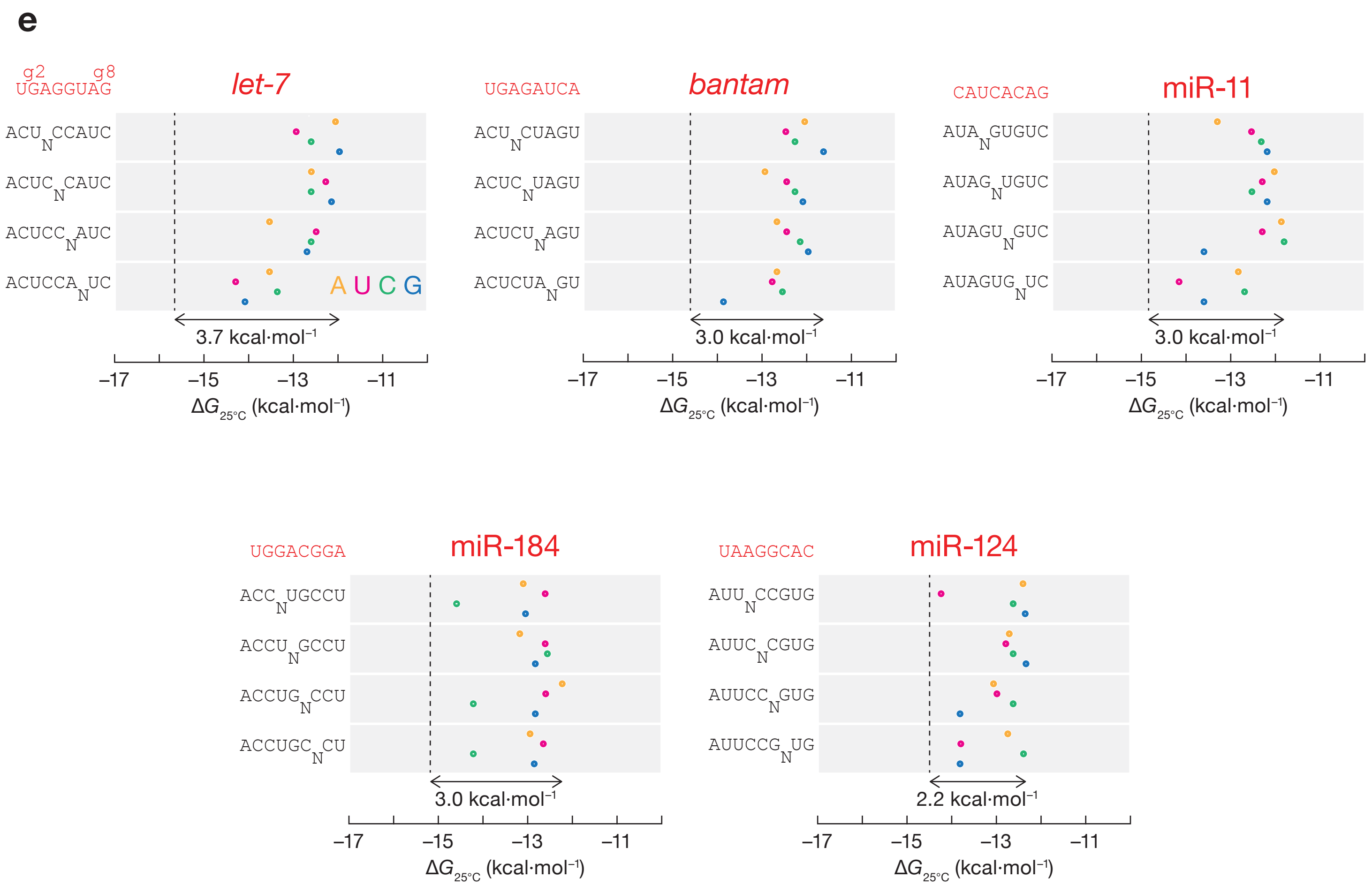

Vega-Badillo et al., Supplementary Figure 4

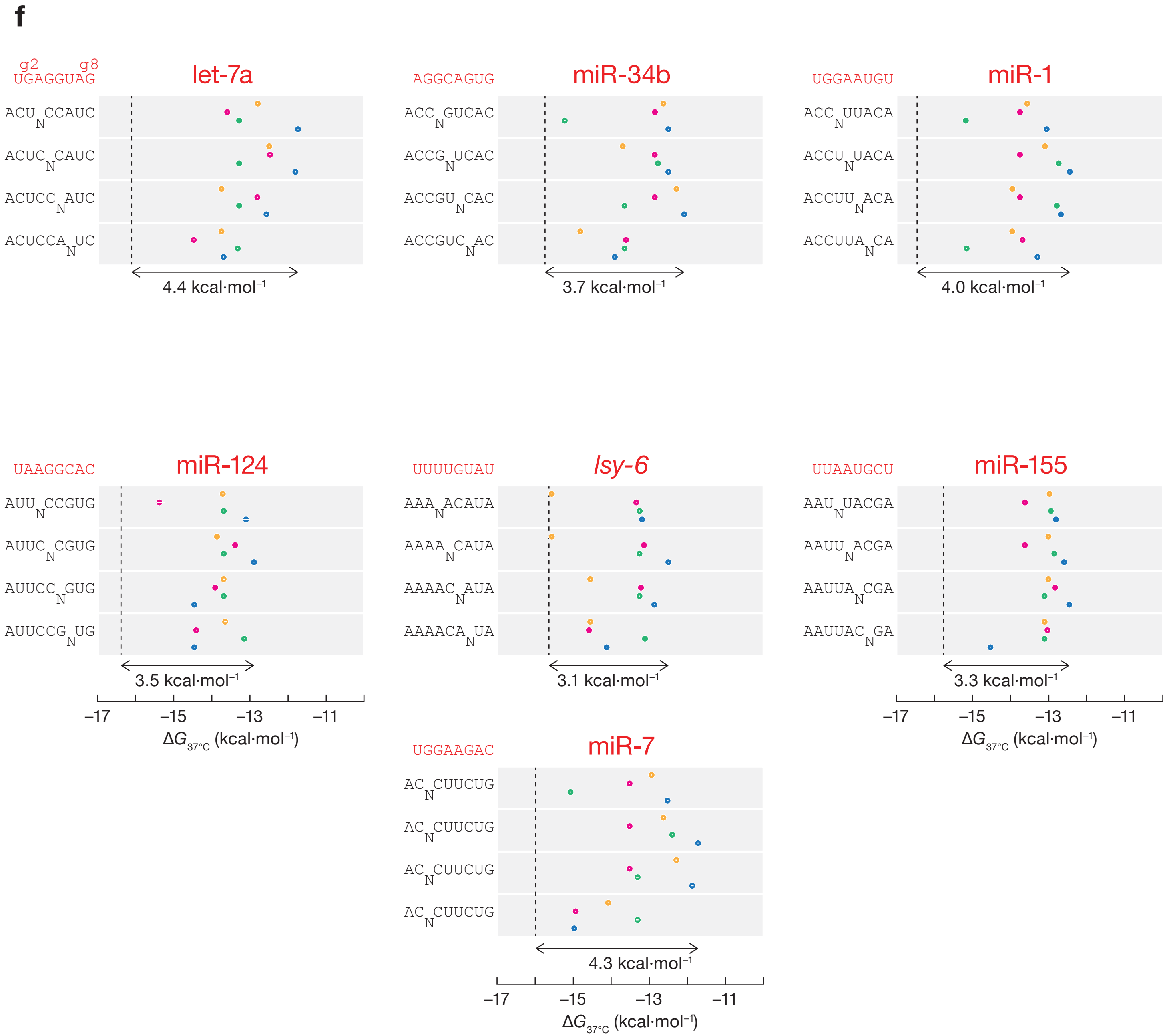

Supplementary Figure 5 | miRNA binding at canonical and noncanonical sites. **(a)** Site types identified by de novo site discovery in *bantam* RBNS and their fitted dissociation constants. Left panel: pairing of each site, indicating Watson–Crick pairing (black) and t1A (blue). Binding experiments were performed with 3.5 mM (upper panel) or 0.89 mM (lower panel) Mg^{2+}. **(b)** Dissociation constants for 3′-only sites. Horizontal dashed lines indicate K_D^{8mer}, K_D^{6mer}, and $K_D^{6mer\text{-}m8}$. Error bars indicate 95% confidence intervals of the median. **(c–d)** Ago1 cleaves centered sites in vitro. **(c)** Uncropped image of polyacrylamide gel electrophoresis shown in Fig. 5b. Sequence-specific cleavage was blocked when the target RNA contained a phosphorothioate linkage (orange) flanked by 2′-*O*-methyl ribose at positions t10 and t11 (cyan). **(d)** Ago1•*let-7* cleaved both centered sites at levels comparable to those observed with a fully complementary RNA. Cleavage reactions were performed with 3.5 nM Mg^{2+} and contained 0.32 nM Ago1•*let-7* and 5 nM **(c)** or 100 nM **(d)** RNA target. Source data are provided as a Source Data file.

Vega-Badillo et al., Supplementary Figure 5

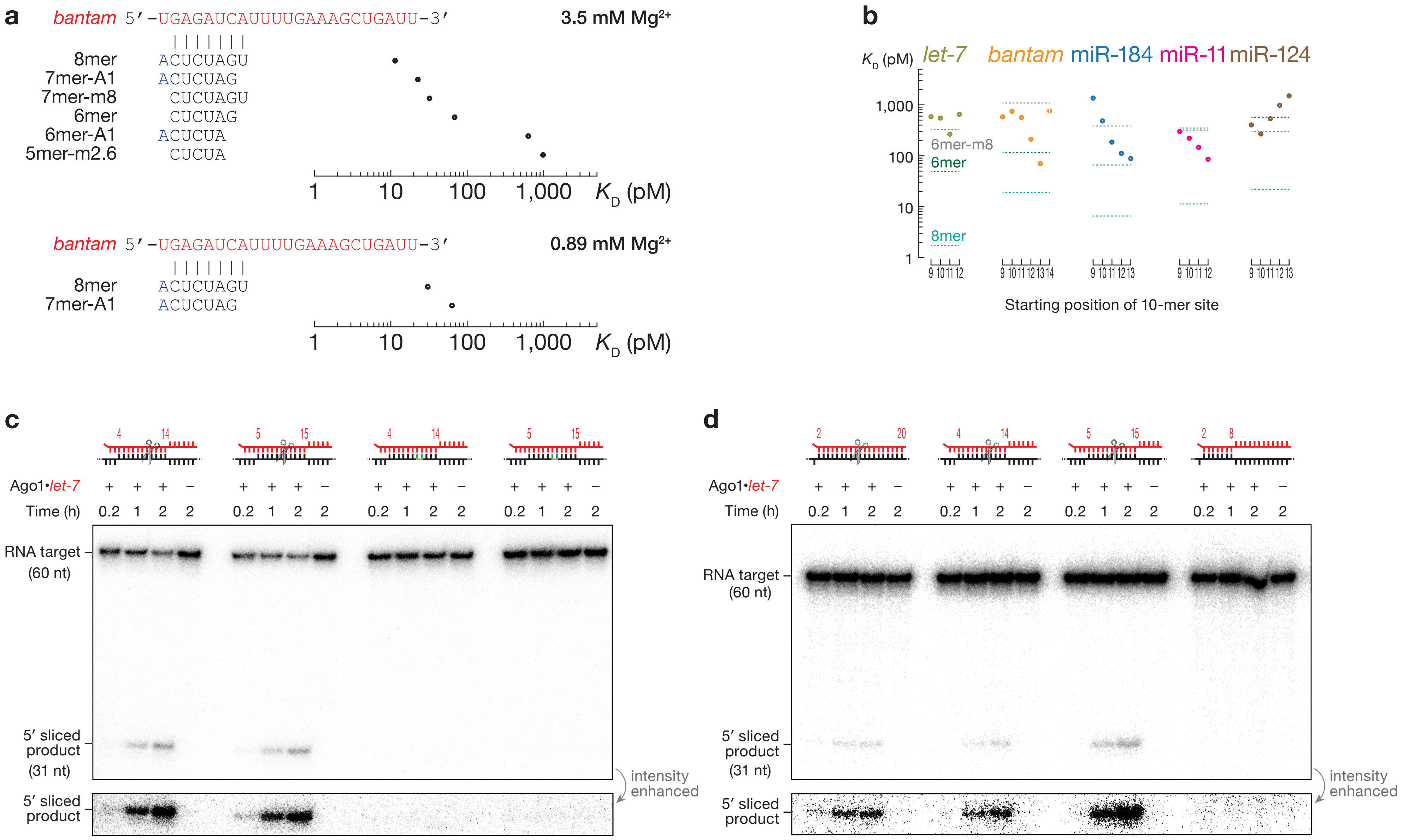

Supplementary Table 1 | RNA and DNA oligonucleotides used in this study

**Ago1 loading**	**Sequence**<br>Seed; p indicates 5′ monophosphate
let7 guide strand	pUGA GGU AGU AGG UUG UAU AGU
let7 passenger strand	UAU ACA AUG UGC UAG CUU UCU
bantam guide strand	pUGA GAU CAU UUU GAA AGC UGA UU
bantam passenger strand	CCG GUU UUC GAU UUG GUU UGA CU
miR-184 guide strand	pUGG ACG GAG AAC UGA UAA GGG C
miR-184 passenger strand	CCU UAU CAU UCU CUC GCC CCG
miR-11 guide strand	pCAU CAC AGU CUG AGU UCU UGC
miR-11 passenger strand	CAA GAA CUU UCU CUG UGA CCC G
miR-124 guide strand	pUAA GGC ACG CGG UGA AUG CCA AG
miR-124 passenger strand	GGU AUC CAC UGU AGG CCU AUA UG
**Ago1·miRNA purification**	**Sequence**<br>RNA, DNA; m, 2′-*O*-methyl ribose; p, 5′ phosphate;<br>Bio, Biotin-6-carbon spacer
Capture Oligo to affinity purify Ago1•*let-7*	Bio-mAmUmA mGmAmC mUmGmC mGmAmC mAmAmU mAmGmC mCmUmA mCmCmU mCmCmG mAmAmC mG
DNA competitor to elute Ago1•*let-7*	Bio-CGT TCG GAG GTA GGC TAT TGT CGC AGT CTA T
Capture Oligo to affinity purify Ago1•*bantam*	Bio-mAmUmA mGmAmC mAmCmU mUmGmU mUmUmC mCmCmU mUmUmG mAmUmC mUmCmC mGmCmC mCmG
DNA competitor to elute Ago1•*bantam*	Bio-CGG GCG GAG ATC AAA GGG AAA CAA GTG TCT AT
Capture Oligo to affinity purify Ago1•miR-184	Bio-mAmUmA mGmUmA mAmAmC mCmAmU mCmAmU mCmCmA mUmCmC mGmUmC mCmCmG mAmAmC mG
DNA competitor to elute Ago1•miR-184	Bio-CGT TCG GGA CGG ATG GAT GAT GGT TTA CTA T
Capture Oligo to affinity purify Ago1•miR-11	Bio-mAmAmA mAmAmA mCmCmU mAmAmC mUmAmU mAmCmC mUmGmU mGmAmU mAmAmA mAmAmG
DNA competitor to elute Ago1•miR-11	Bio-CTT TTT ATC ACA GGT ATA GTT AGG TTT TTT
Capture Oligo to affinity purify Ago1•miR-124	Bio-mAmUmA mGmAmC mAmUmA mAmUmU mUmCmA mAmAmA mAmGmU mGmCmC mUmUmC mGmAmA mCmG
DNA competitor to elute Ago1•miR-124	Bio-CGT TCG AAG GCA CTT TTT GAA ATT ATG TCT AT

**Ago1•miRNA quantification**	**Sequence**<br>p, 5′ phosphate
RNA probe to quantify total concentration of Ago1•*let-7* by Northern Blot	pGAU ACU AUA CAA CCU ACU ACC UCA ACC U
RNA probe to quantify total concentration of Ago1•*bantam* by Northern Blot	pGUU AAU CAG CUU UCA AAA UGA UCU CAU AGA
RNA probe to quantify total concentration of Ago1•miR-184 by Northern Blot	pGUA GCC CUU AUC AGU UCU CCG UCC AAU UA
RNA probe to quantify total concentration of Ago1•miR-11 by Northern Blot	pGUA GCA AGA ACU CAG ACU GUG AUG AAG U
RNA probe to quantify total concentration of Ago1•miR-124 by Northern Blot	pGAU CUU GGC AUU CAC CGC GUG CCU UAA CCU
RNA target to quantify active concentration of Ago1•*let-7* by double-filter binding	pGAA AAA AAA AAA AAA UCU ACC UCU AAA U
RNA target to quantify active concentration of Ago1•*bantam* by double-filter binding	pAAA AAA AAA AAA AUU UUU GAU CUC UAA AU
RNA target to quantify active concentration of Ago1•miR-184 by double-filter binding	pGAA AAA AAA AAA AAA AAU CCG UCC UAA AU
RNA target to quantify active concentration of Ago1•miR-11 by double-filter binding	pGAA AAU UUU AAA AAA UCU GUG AUA AAA U
RNA target to quantify active concentration of Ago1•miR-124 by double-filter binding	pGAA AAA AAA UAA AUA AAA GUG CCU UUA AAU

**Substrates for competition assays**	**Sequence** p, 5′ phosphate; m, 2′-*O*-methyl ribose; ps, phosphorothioate; (Pu), Purine ribonucleoside; complementary to guide
Target to *let-7* with g2–21 complementarity	pGAU ACU AUA CAA CmCpsmU ACU ACC UCA ACC U
Target to *let-7* with g2–21 complementarity and a G:U pair at position 4	pGAU ACU AUA CAA CmCpsmU ACU ACU UCA ACC U
Target to *let-7* with g2–21 complementarity and G:U pairs at positions 4–5	pGAU ACU AUA CAA CmCpsmU ACU AUU UCA ACC U
Target to *let-7* with g2–8 complementarity and mismatches at positions 4–5	pGAA AAA AAA AAA AmApsmA UCU AAA UCA AAA U
Target to *let-7* with g2–8 and g13–16 complementarity and mismatches at positions 4–5	pGAA AAA AAA CAA AmApsmA UCU AAA UCA AAA U
Target to *let-7* with g2–8 and g12–17 complementarity and mismatches at positions 4–5	pGAA AAA UUA CAA CmApsmA UCU AAA UCA AAA U
Target to *let-7* with g2–21 complementarity and mismatches at positions 4–5	pGAU ACU AUA CAA CmCpsmU ACU AAA UCA ACC U
30-nt poly(A) RNA	pAAA AAA AAA AAA AAA AAA AAA AAA AAA AAA
Unnatural 30-nt poly(A) RNA	pAAA (Pu)AAA (Pu)AAA (Pu)AAA (Pu)AAA (Pu)AAA (Pu)AAA (Pu)AA
**Substrates for in vitro cleavage**	**Sequence** Seed; m, 2′-*O*-methyl ribose; ps, phosphorothioate; complementary to guide
Target to *let-7* with g2–20 complementarity	GAG UUC UAC AGU CCG ACG AUC CUA UAC AAC CUA CUA CCU CAU GGA AUU CUC GGG UGC CAA
Target to *let-7* with g2–8 complementarity	GAG UUC UAC AGU CCG ACG AUC AAA AAA AAA AAU CUA CCU CAU GGA AUU CUC GGG UGC CAA
Target to *let-7* with g4–14 complementarity	GAG UUC UAC AGU CCG ACG AUC AAA AAA AAC CUA CUA CCA AUU GGA AUU CUC GGG UGC CAA
Target to *let-7* with g5–15 complementarity	GAG UUC UAC AGU CCG ACG AUC AAA AUC AAC CUA CUA CAA AUU GGA AUU CUC GGG UGC CAA
Target to *let-7* with g4–14 complementarity and modified t10,t11	GAG UUC UAC AGU CCG ACG AUC AAA AAA AAC mCpsmUA CUA CCA AUU GGA AUU CUC GGG UGC CAA
Target to *let-7* with g5–15 complementarity and modified t10,t11	GAG UUC UAC AGU CCG ACG AUC AAA AUC AAC mCpsmUA CUA CAA AUU GGA AUU CUC GGG UGC CAA

RBNS	**Sequence**<br>RNA, DNA
RBNS RNA input pool	pGAG UUC UAC AGU CCG ACG AUC NNN NNN NNN NNN NNN NNN NNU GGA AUU CUC GGG UGC CAA
5′-end blocking cDNA oligonucleotide #1	GTC GGA CTG TAG AAC TC
3′-end blocking cDNA oligonucleotide #1	TTG GCA CCC GAG AAT
5′-end blocking cDNA oligonucleotide #2	CGG ACT GTA GAA CTC
3′-end blocking cDNA oligonucleotide #2	TTG GCA CCC GAG A
RT primer	CCT TGG CAC CCG AGA ATT CCA
PCR Forward primer	AAT GAT ACG GCG ACC ACC GAG ATC TAC ACG TTC AGA GTT CTA CAG TCC GA
Multiplexing PCR Reverse Primer PCRId1	CAA GCA GAA GAC GGC ATA CGA GAT CGT GAT GTG ACT GGA GTT CCT TGG CAC CCG AGA ATT CCA
Multiplexing PCR Reverse Primer PCRId2	CAA GCA GAA GAC GGC ATA CGA GAT ACA TCG GTG ACT GGA GTT CCT TGG CAC CCG AGA ATT CCA
Multiplexing PCR Reverse Primer PCRId3	CAA GCA GAA GAC GGC ATA CGA GAT GCC TAA GTG ACT GGA GTT CCT TGG CAC CCG AGA ATT CCA
Multiplexing PCR Reverse Primer PCRId4	CAA GCA GAA GAC GGC ATA CGA GAT TGG TCA GTG ACT GGA GTT CCT TGG CAC CCG AGA ATT CCA
Multiplexing PCR Reverse Primer PCRId5	CAA GCA GAA GAC GGC ATA CGA GAT CAC TGT GTG ACT GGA GTT CCT TGG CAC CCG AGA ATT CCA
Multiplexing PCR Reverse Primer PCRId6	CAA GCA GAA GAC GGC ATA CGA GAT ATT GGC GTG ACT GGA GTT CCT TGG CAC CCG AGA ATT CCA
Multiplexing PCR Reverse Primer PCRId7	CAA GCA GAA GAC GGC ATA CGA GAT GAT CTG GTG ACT GGA GTT CCT TGG CAC CCG AGA ATT CCA
Multiplexing PCR Reverse Primer PCRId8	CAA GCA GAA GAC GGC ATA CGA GAT TCA AGT GTG ACT GGA GTT CCT TGG CAC CCG AGA ATT CCA
Multiplexing PCR Reverse Primer PCRId9	CAA GCA GAA GAC GGC ATA CGA GAT CTG ATC GTG ACT GGA GTT CCT TGG CAC CCG AGA ATT CCA
Multiplexing PCR Reverse Primer PCRId10	CAA GCA GAA GAC GGC ATA CGA GAT AAG CTA GTG ACT GGA GTT CCT TGG CAC CCG AGA ATT CCA
Multiplexing PCR Reverse Primer PCRId11	CAA GCA GAA GAC GGC ATA CGA GAT GTA GCC GTG ACT GGA GTT CCT TGG CAC CCG AGA ATT CCA

Multiplexing PCR Reverse Primer PCRId12	CAA GCA GAA GAC GGC ATA CGA GAT TAC AAG GTG ACT GGA GTT CCT TGG CAC CCG AGA ATT CCA
Multiplexing PCR Reverse Primer PCRId13	CAA GCA GAA GAC GGC ATA CGA GAT TTG ACT GTG ACT GGA GTT CCT TGG CAC CCG AGA ATT CCA
Multiplexing PCR Reverse Primer PCRId14	CAA GCA GAA GAC GGC ATA CGA GAT GGA ACT GTG ACT GGA GTT CCT TGG CAC CCG AGA ATT CCA
Multiplexing PCR Reverse Primer PCRId15	CAA GCA GAA GAC GGC ATA CGA GAT TGA CAT GTG ACT GGA GTT CCT TGG CAC CCG AGA ATT CCA
Multiplexing PCR Reverse Primer PCRId16	CAA GCA GAA GAC GGC ATA CGA GAT GGA CGG GTG ACT GGA GTT CCT TGG CAC CCG AGA ATT CCA
Multiplexing PCR Reverse Primer PCRId17	CAA GCA GAA GAC GGC ATA CGA GAT CTC TAC GTG ACT GGA GTT CCT TGG CAC CCG AGA ATT CCA
Multiplexing PCR Reverse Primer PCRId18	CAA GCA GAA GAC GGC ATA CGA GAT GCG GAC GTG ACT GGA GTT CCT TGG CAC CCG AGA ATT CCA
Multiplexing PCR Reverse Primer PCRId19	CAA GCA GAA GAC GGC ATA CGA GAT TTT CAC GTG ACT GGA GTT CCT TGG CAC CCG AGA ATT CCA
Multiplexing PCR Reverse Primer PCRId20	CAA GCA GAA GAC GGC ATA CGA GAT GGC CAC GTG ACT GGA GTT CCT TGG CAC CCG AGA ATT CCA
Multiplexing PCR Reverse Primer PCRId21	CAA GCA GAA GAC GGC ATA CGA GAT CGA AAC GTG ACT GGA GTT CCT TGG CAC CCG AGA ATT CCA
Multiplexing PCR Reverse Primer PCRId22	CAA GCA GAA GAC GGC ATA CGA GAT CGT ACG GTG ACT GGA GTT CCT TGG CAC CCG AGA ATT CCA
Multiplexing PCR Reverse Primer PCRId23	CAA GCA GAA GAC GGC ATA CGA GAT CCA CTC GTG ACT GGA GTT CCT TGG CAC CCG AGA ATT CCA
Multiplexing PCR Reverse Primer PCRId24	CAA GCA GAA GAC GGC ATA CGA GAT GCT ACC GTG ACT GGA GTT CCT TGG CAC CCG AGA ATT CCA
Multiplexing PCR Reverse Primer PCRId25	CAA GCA GAA GAC GGC ATA CGA GAT ATC AGT GTG ACT GGA GTT CCT TGG CAC CCG AGA ATT CCA
Multiplexing PCR Reverse Primer PCRId26	CAA GCA GAA GAC GGC ATA CGA GAT GCT CAT GTG ACT GGA GTT CCT TGG CAC CCG AGA ATT CCA
Multiplexing PCR Reverse Primer PCRId27	CAA GCA GAA GAC GGC ATA CGA GAT AGG AAT GTG ACT GGA GTT CCT TGG CAC CCG AGA ATT CCA
Multiplexing PCR Reverse Primer PCRId28	CAA GCA GAA GAC GGC ATA CGA GAT CTT TTG GTG ACT GGA GTT CCT TGG CAC CCG AGA ATT CCA

Multiplexing PCR Reverse Primer PCRId29	CAA GCA GAA GAC GGC ATA CGA GAT TAG TTG GTG ACT GGA GTT CCT TGG CAC CCG AGA ATT CCA
Multiplexing PCR Reverse Primer PCRId30	CAA GCA GAA GAC GGC ATA CGA GAT CCG GTG GTG ACT GGA GTT CCT TGG CAC CCG AGA ATT CCA
Multiplexing PCR Reverse Primer PCRId31	CAA GCA GAA GAC GGC ATA CGA GAT ATC GTG GTG ACT GGA GTT CCT TGG CAC CCG AGA ATT CCA
Multiplexing PCR Reverse Primer PCRId32	CAA GCA GAA GAC GGC ATA CGA GAT TGA GTG GTG ACT GGA GTT CCT TGG CAC CCG AGA ATT CCA
Multiplexing PCR Reverse Primer PCRId33	CAA GCA GAA GAC GGC ATA CGA GAT CGC CTG GTG ACT GGA GTT CCT TGG CAC CCG AGA ATT CCA
Multiplexing PCR Reverse Primer PCRId34	CAA GCA GAA GAC GGC ATA CGA GAT GCC ATG GTG ACT GGA GTT CCT TGG CAC CCG AGA ATT CCA
Multiplexing PCR Reverse Primer PCRId35	CAA GCA GAA GAC GGC ATA CGA GAT AAA ATG GTG ACT GGA GTT CCT TGG CAC CCG AGA ATT CCA
Multiplexing PCR Reverse Primer PCRId36	CAA GCA GAA GAC GGC ATA CGA GAT TGT TGG GTG ACT GGA GTT CCT TGG CAC CCG AGA ATT CCA
**Filter binding assay for Ago1·let-7**	**Sequence**<br>Seed; m, 2′-*O*-methyl ribose; ps, phosphorothioate<br>complementary to guide
Complete complementary target to *let-7*	pGAUACUAUACAACmCpsmUACUACCUCAACCU
Target to *let-7* with seed only pairing (g2g8:t2–t8)	pGAAAAAAAAAAAAmApsmAUCUACCUCUAAAU
