# Supplementary material for: Biochemical principles of miRNA targeting in flies
Source: Nat Commun. 2026 Jan 20;17:1641. doi: 10.1038/s41467-026-68360-0 (PMC12905440; doi:10.1038/s41467-026-68360-0)
Supplement: Supplementary file 2 — Description of Additional Supplementary Files [file 41467_2026_68360_MOESM2_ESM.pdf]

Description of Additional Supplementary Files

Supplementary Data 1: Maximum likelihood estimates of miRISC K_D values at convergence. Units for all parameters are nanomolar (nM), except for ƒcost, which is expressed in arbitrary units.
