# Supplementary material for: Biochemical principles of miRNA targeting in flies
Source: Nat Commun. 2026 Jan 20;17:1641. doi: 10.1038/s41467-026-68360-0 (PMC12905440; doi:10.1038/s41467-026-68360-0)
Supplement: Supplementary file 4 — Reporting Summary [file 41467_2026_68360_MOESM4_ESM.pdf]

nature portfolio

Corresponding author(s): Karina Jouravleva, Joel Vega-Badillo

Last updated by author(s): 2025/12/10

Reporting Summary

Nature Portfolio wishes to improve the reproducibility of the work that we publish. This form provides structure for consistency and transparency in reporting. For further information on Nature Portfolio policies, see our Editorial Policies and the Editorial Policy Checklist.

Statistics

For all statistical analyses, confirm that the following items are present in the figure legend, table legend, main text, or Methods section.

n/a	Confirmed	
☐	☒	The exact sample size (*n*) for each experimental group/condition, given as a discrete number and unit of measurement
☐	☒	A statement on whether measurements were taken from distinct samples or whether the same sample was measured repeatedly
☐	☒	The statistical test(s) used AND whether they are one- or two-sided<br>*Only common tests should be described solely by name; describe more complex techniques in the Methods section.*
☐	☒	A description of all covariates tested
☐	☒	A description of any assumptions or corrections, such as tests of normality and adjustment for multiple comparisons
☐	☒	A full description of the statistical parameters including central tendency (e.g. means) or other basic estimates (e.g. regression coefficient) AND variation (e.g. standard deviation) or associated estimates of uncertainty (e.g. confidence intervals)
☐	☒	For null hypothesis testing, the test statistic (e.g. *F*, *t*, *r*) with confidence intervals, effect sizes, degrees of freedom and *P* value noted<br>*Give P values as exact values whenever suitable.*
☒	☐	For Bayesian analysis, information on the choice of priors and Markov chain Monte Carlo settings
☒	☐	For hierarchical and complex designs, identification of the appropriate level for tests and full reporting of outcomes
☐	☒	Estimates of effect sizes (e.g. Cohen's *d*, Pearson's *r*), indicating how they were calculated

Our web collection on statistics for biologists contains articles on many of the points above.

Software and code

Policy information about availability of computer code

Data collection	Typhoon FLA 7000, Illumina NextSeq 500, Illumina NextSeq 2000
Data analysis	IgorPro 6.11 (WaveMetrics), IgorPro9.05 (WaveMetrics), Microsoft Excel Version 16.90.2, ImageQuant TL, ImageJ 1.54g, Python2

For manuscripts utilizing custom algorithms or software that are central to the research but not yet described in published literature, software must be made available to editors and reviewers. We strongly encourage code deposition in a community repository (e.g. GitHub). See the Nature Portfolio guidelines for submitting code & software for further information.

Data

Policy information about availability of data

All manuscripts must include a data availability statement. This statement should provide the following information, where applicable:

- Accession codes, unique identifiers, or web links for publicly available datasets
- A description of any restrictions on data availability
- For clinical datasets or third party data, please ensure that the statement adheres to our policy

RBNS sequencing data have been deposited at National Center for Biotechnology Information Sequence Read Archive and are publicly available using accession number PRJNA1185003. Source Data are provided with this paper.

Research involving human participants, their data, or biological material

Policy information about studies with human participants or human data. See also policy information about sex, gender (identity/presentation), and sexual orientation and race, ethnicity and racism.

Reporting on sex and gender	N/A
Reporting on race, ethnicity, or other socially relevant groupings	N/A
Population characteristics	N/A
Recruitment	N/A
Ethics oversight	N/A

Note that full information on the approval of the study protocol must also be provided in the manuscript.

Field-specific reporting

Please select the one below that is the best fit for your research. If you are not sure, read the appropriate sections before making your selection.

☒ Life sciences ☐ Behavioural & social sciences ☐ Ecological, evolutionary & environmental sciences

For a reference copy of the document with all sections, see nature.com/documents/nr-reporting-summary-flat.pdf

Life sciences study design

All studies must disclose on these points even when the disclosure is negative.

Sample size	Competition and direct binding assays: 3 independent trials. Cleavage assays for central sites: 3 independent trials. MLE of KD values: 2 independent trials, bootstrapped 10 times, 100 different combinations of 10 initial guesses of estimated parameters.
Data exclusions	No data were excluded from the analysis
Replication	All data were collected during independent trials conducted on separate days.
Randomization	Biochemical experiments are rarely randomized. Therefore, randomization is not relevant to this study.
Blinding	Biochemical experiments are rarely blinded. Therefore, blinding was not performed during data acquisition

Reporting for specific materials, systems and methods

We require information from authors about some types of materials, experimental systems and methods used in many studies. Here, indicate whether each material, system or method listed is relevant to your study. If you are not sure if a list item applies to your research, read the appropriate section before selecting a response.

Materials & experimental systems

n/a	Involved in the study	
☒	☐	Antibodies
☐	☒	Eukaryotic cell lines
☒	☐	Palaeontology and archaeology
☐	☒	Animals and other organisms
☒	☐	Clinical data
☒	☐	Dual use research of concern
☒	☐	Plants

Methods

n/a	Involved in the study	
☒	☐	ChIP-seq
☒	☐	Flow cytometry
☒	☐	MRI-based neuroimaging

Eukaryotic cell lines

Policy information about cell lines and Sex and Gender in Research

Cell line source(s)	Drosophila Schneider 2 (S2) cells (Life Technologies, catalog no. R690-07)

Authentication	Cells were purchased from Life Technologies and used only to produce recombinant proteins.
Mycoplasma contamination	Cells were tested and were negative for Mycoplasma contamination.
Commonly misidentified lines (See ICLAC register)	No commonly misidentified cell lines were used in the study

Animals and other research organisms

Policy information about studies involving animals; ARRIVE guidelines recommended for reporting animal research, and Sex and Gender in Research

Laboratory animals	Fly strains: Description w[*]; AGO2[414]); Stock Number (109027); Collection (Kyoto Stock Center); FlyBase ID (FBst0313641)
Wild animals	N/A
Reporting on sex	N/A
Field-collected samples	N/A
Ethics oversight	Not required: this study used Drosophila melanogaster and did not involve vertebrates or human subjects.

Note that full information on the approval of the study protocol must also be provided in the manuscript.

Plants

Seed stocks	N/A
Novel plant genotypes	N/A
Authentication	N/A
